# Multimodal Evaluation of TMS - Induced Somatosensory Plasticity and Behavioral Recovery in Rats With Contusion Spinal Cord Injury

**DOI:** 10.3389/fnins.2019.00387

**Published:** 2019-04-24

**Authors:** Vijai S. Krishnan, Samuel S. Shin, Visar Belegu, Pablo Celnik, Mark Reimers, Kylie R. Smith, Galit Pelled

**Affiliations:** ^1^Department of Biomedical Engineering, Michigan State University, East Lansing, MI, United States; ^2^The Institute for Quantitative Health Science and Engineering, Michigan State University, East Lansing, MI, United States; ^3^F.M. Kirby Research Center for Functional Brain Imaging, Kennedy Krieger Institute, Baltimore, MD, United States; ^4^Russell H. Morgan Department of Radiology and Radiological Science, Johns Hopkins University School of Medicine, Baltimore, MD, United States; ^5^Department of Neurology and Pathology, Johns Hopkins University School of Medicine, Baltimore, MD, United States; ^6^International Center for Spinal Cord Injury, Kennedy Krieger Institute, Baltimore, MD, United States; ^7^Department of Physical Medicine and Rehabilitation, Johns Hopkins University School of Medicine, Baltimore, MD, United States; ^8^Department of Radiology, Michigan State University, East Lansing, MI, United States

**Keywords:** transcranial magnetic stimulation, spinal cord injury, plasticity, behavior, functional magnetic resonance imaging

## Abstract

**Introduction:** Spinal cord injury (SCI) causes partial or complete damage to sensory and motor pathways and induces immediate changes in cortical function. Current rehabilitative strategies do not address this early alteration, therefore impacting the degree of neuroplasticity and subsequent recovery. The following study aims to test if a non-invasive brain stimulation technique such as repetitive transcranial magnetic stimulation (rTMS) is effective in promoting plasticity and rehabilitation, and can be used as an early intervention strategy in a rat model of SCI.

**Methods:** A contusion SCI was induced at segment T9 in adult rats. An rTMS coil was positioned over the brain to deliver high frequency stimulation. Behavior, motor and sensory functions were tested in three groups: SCI rats that received high-frequency (20 Hz) rTMS within 10 min post-injury (acute-TMS; *n* = 7); SCI rats that received TMS starting 2 weeks post-injury (chronic-TMS; *n* = 5), and SCI rats that received sham TMS (no-TMS, *n* = 5). Locomotion was evaluated by the Basso, Beattie, and Bresnahan (BBB) and gridwalk tests. Motor evoked potentials (MEP) were recorded from the forepaw across all groups to measure integrity of motor pathways. Functional MRI (fMRI) responses to contralateral tactile hindlimb stimulation were measured in an 11.7T horizontal bore small-animal scanner.

**Results:** The acute-TMS group demonstrated the fastest improvements in locomotor performance in both the BBB and gridwalk tests compared to chronic and no-TMS groups. MEP responses from forepaw showed significantly greater difference in the inter-peak latency between acute-TMS and no-TMS groups, suggesting increases in motor function. Finally, the acute-TMS group showed increased fMRI-evoked responses to hindlimb stimulation over the right and left hindlimb (LHL) primary somatosensory representations (S1), respectively; the chronic-TMS group showed moderate sensory responses in comparison, and the no-TMS group exhibited the lowest sensory responses to both hindlimbs.

**Conclusion:** The results suggest that rTMS therapy beginning in the acute phase after SCI promotes neuroplasticity and is an effective rehabilitative approach in a rat model of SCI.

## Introduction

Traumatic spinal cord injury (SCI) is a debilitating condition with a reported global incidence ranging from 10 to 80 million per population per year (Singh et al., 2014; Jazayeri et al., 2015). The changes in the cortex and the thalamus following SCI have been studied extensively (Bonatz et al., 2000; Aguilar et al., 2010; Ghosh et al., 2010, 2012). Deafferentation caused by SCI has been shown to induce short (days) and long-term (months) alterations in brain architecture and neuronal connections (Jain et al., 2008; Ghosh et al., 2010). However, it is still unclear how these changes translate into behavior and recovery.

Evidence from rodent models suggest that within minutes after SCI, decreases in spontaneous neuronal activity are observed in cortical areas that correspond to the injured limbs as well as cortical areas that normally process information from the non-injured limbs (Aguilar et al., 2010). Moreover, these decreases in spontaneous activity are correlated with poor recovery. Thus, an intervention to attenuate and reverse the injury-induced reduction in spontaneous cortical activity has the potential to accelerate post-SCI neurorehabilitation. Indeed, there are ongoing efforts to modulate the activity of the peripheral and central nervous system after SCI with the goal of enhancing recovery. A bulk of evidence demonstrates that peripheral nerve function is affected by SCI (Boland et al., 2010; Van De Meent et al., 2010) highlighting the importance of regulating peripheral nerve function during critical phases of SCI. For example, an intensive 6 week peripheral nerve stimulation regimen has been shown to prevent long term changes in axonal function post-SCI (Lee et al., 2015). Other non-pharmacological and non-invasive approaches such as spinal cord stimulation (functional electrical stimulation) (Martin et al., 2012) has been shown to improve recovery outcomes post-SCI. Functional electrical stimulation of central pattern generator mechanisms has been shown to improve responses in patients with complete or incomplete SCI (Harkema et al., 2011). Recent research has shown that the application of epidural electric stimulation (EES) improves spinal networks post-injury through the restoration of locomotion (Courtine et al., 2009; Formento et al., 2018). The future of neurorehabilitative strategies would involve using non-invasive treatments involving electrical stimulation.

In addition to assessing corticospinal transmission times, non-invasive brain stimulation technologies are additionally being used as therapies for a variety of neurological disorders and diseases (Schulz et al., 2013; Gunduz et al., 2017). A prominent FDA approved (O’Reardon et al., 2007), non-invasive technology shown to produce long lasting increases in cortical excitability is transcranial magnetic stimulation (TMS). TMS has been shown to induce neuronal excitation and plasticity beyond the stimulation period in several injury and disease conditions (Lefaucheur et al., 2014; Shin and Pelled, 2017), including in rodent models of brain injury (Lu et al., 2015; Shin et al., 2018). Reports in the past have demonstrated that applying TMS years after SCI has improved motor and sensory outcomes (Belci et al., 2004; Kuppuswamy et al., 2011). Another study showed that high-frequency repetitive TMS (rTMS) stimulation applied months after SCI improved motor function compared to the same treatment with sham stimulation (Benito et al., 2012).

This led us to test whether applying TMS over the cortex to enhance excitation, within days and weeks after the injury was sustained, could facilitate recovery in an animal model.

A contusion model of thoracic injury (T9) in adult rats shown to mimic similar pathological changes to human SCI (Metz et al., 2000a) was used. We tested if daily TMS sessions starting within minutes or weeks after the injury would accelerate neuroplasticity. MEP was used to test the effectiveness of TMS treatment by evaluating the changes in integrity of motor pathways (Curt et al., 1998; Nakamae et al., 2010). Weekly behavioral testing was conducted to assess gross behavior improvement. High-resolution functional MRI was used to detect cortical functional responses evoked by tactile limb stimulation (Pelled et al., 2006, 2009; Han et al., 2013; Li et al., 2014b; Lu et al., 2015) as a means to evaluate recovery of ascending spinal pathways.

Our results support the hypothesis that rescuing the hypoactive neuronal activity by TMS can accelerate post-SCI neuroplasticity.

## Materials and Methods

All animal procedures were conducted in accordance with the NIH *Guide for the Care and Use of Laboratory Animals* and approved by the Johns Hopkins University Animal Care and Use Committee.

### Animals

17 male adult Sprague-Dawley rats (Harlan Laboratories) were provided with food and water *ad libitum* and housed in pairs (standard housing).

### Spinal Cord Injury

An hour prior to surgery, rats were injected with buprenorphine (Buprenex; 0.05 mg/kg, s.c.). Animals were anesthetized using a Ketamine (75 mg/kg, i.p.) and Dexmedetomidine (0.25 mg/kg, i.p.) cocktail. An infinite horizon impactor was used to induce SCI. Displacement and velocity were tested to ensure instrument reliability (Scheff et al., 2003). A midline sagittal incision centered over the T9 vertebra was performed, and the muscles and connective tissue were separated to expose the spinal segments. Dorsal laminectomy of the T9 segment was followed. Afterward, adson forceps was used to clamp the spinal column, rostral and caudal to the laminectomy. The animal was transferred to the IH impactor and was centered with the exposed site immediately beneath the impactor. The impactor was lowered with a 4 mm tip above the exposed spinal cord segment. A 200 kdyne injury was used to impact the exposed segment, resulting in a severe contusion SCI (Cao et al., 2005; Anderson et al., 2009).

After the injury the muscle and fascia were sutured with a running 5–0 vicryl absorbable suture and the skin was closed using wound clips. Rats were given 10 ml of Ringers solution (i.p), 0.05 mL of gentamicin (i.m) and buprenorphine (Buprenex; 0.05 mg/kg, s.c.), and put in a 37°C incubator. Once in the incubator, Antisedan (1 mg/kg; i.m.) was administered to reverse the anesthesia. Water Gel pack and food pellets were provided at the bottom of the cage up to 72 h after SCI. Bladder expression was performed twice daily until the animals regained bladder control. Buprenorphine was administered twice a day for 48 h. Administration of 10 mL of Ringer’s solution (i.p.) and 0.05 mL of gentamicin (i.m.) continued daily for 7 days. Wound clips were removed 7–10 days after injury. Animals showing lack of movement were not included in this study.

### Transcranial Magnetic Stimulation

For TMS application, rats were anesthetized with 2% isoflurane and their heads secured using a stereotaxic frame. The TMS system (Magstim, Rapid2) was equipped with a figure eight, 25 mm custom rodent coil which was placed over the midline between bregma and lambda, covering both left and right sensorimotor cortices. The stimulus was delivered 3-times a week for 6 weeks using the following settings: 4 s cycles of 10 Hz stimuli, 26 s interval between cycles, and 7 cycles (total of 280 pulses per day, 1680 total stimuli). The length of this stimulation treatment was 10 min. Injured rats were divided into three groups:***Acute-TMS Group (n = 7)***, TMS delivered within 10 min following SCI and wound suturing;***Chronic-TMS Group (n = 5)***, TMS beginning 2 weeks following SCI; ***No-TMS Group (n = 5)***. The TMS coil was placed exactly in the same position as in the experimental groups but no stimulation was applied. At the end of the procedure the animals were placed in a clean cage on a 37°C warming tray and monitored for return to normal activity.

### Basso, Beattie, and Bresnahan (BBB) Locomotor Scale

In order to assess motor function post-SCI, rats were placed in an uninterrupted open field and allowed unrestricted movement. Rats were allowed to move freely and were scored by their ability to use their hindlimbs. A 21- point BBB locomotion scale was used based on the movement of joints, placement of paws and coordination of forepaw and hindlimbs (Basso et al., 1995).

### Gridwalk Test

Motor behavior was assessed by performing the weekly gridwalk test. The gridwalk test was used to evaluate sensory motor coordination after SCI. In this test, the rats were examined in a long walkway consisting of irregularly spaced metal rungs over which the animals must travel in order to reach the end. The number of foot fall errors where the hindlimb of a test animal failed to grasp a bar and fell between the bars were recorded.

### Electromyography

Motor evoked potentials (MEP) in response to TMS were recorded with needle electrodes inserted into both forepaws. The corticospinal excitability was measured in response to stimulatory TMS at 100% capacity for 10 single stimuli. Inter-peak latency responses to the first TMS stimulus in each rat forepaw were measured using Spike 2 software (CED, Cambridge, United Kingdom). Inter-peak latency was calculated as the difference in time between the two consecutive peaks of the MEP.

### fMRI Acquisition and Data Analysis

Rats were anesthetized with dexmedetomidine (0.1 mg/kg/h, SC) which is known to preserve neurovascular coupling (Li et al., 2014a). Rats were then placed in an ultra-high field 11.7 Tesla/16 cm horizontal bore small-animal scanner (Bruker BioSpin, Rheinstetten, Germany). A 72-mm quadrature volume coil and a 15-mm-diameter surface coil were used to transmit and receive magnetic resonance signals, respectively.

Respiration rate, heart rate, rectal temperature, and partial pressure of oxygen were continuously monitored throughout fMRI measurements (Starr Life Sciences, Pennsylvania, United States). fMRI, gradient echo, echo planar imaging was used with a resolution of 150 × 150 × 1000 μm. Five 1 mm thick coronal slices covering the primary somatosensory cortex (S1) were acquired [effective echo time (TE), 11 ms; repetition time (TR), 1000 ms; bandwidth, 250 KHz; field of view (FOV), 1.92 × 1.92 cm; and matrix size, 128 × 128]. A T2-weighted RARE sequence was used to acquire high-resolution anatomical images (TE, 10 ms; TR, 5000 ms; bandwidth, 250 KHz; FOV, 1.92 × 1.92 cm; and matrix size, 256 × 256) corresponding to the fMRI measurements. Two needle electrodes were inserted into the left and right forepaws or hindlimbs to deliver electrical stimulation. Electrical stimulation (9 Hz, 0.3 mA, and 0.3 ms) was applied for two trains of 20 s with 40 s rest in between. Cross-correlation maps were cluster-size thresholded for an effective significance of *P* < 0.05 using Stimulate (University of Minnesota).

### Statistical Analysis

Statistical analysis was performed using Graphpad Prism package (San Diego, CA, United States). For the BBB scores, gridwalk, and fMRI studies, we used one-way ANOVA with multiple comparisons (followed by sidak *post hoc* test) to verify differences between groups. Linear regression analysis was also used for gridwalk test. MEP data were analyzed using student’s *t*-test. Data are presented as means ± SEM. *P* value of <0.05 was considered significant for all the data analysis. ^∗^, ^∗∗^, and ^∗∗∗^ correspond to *p* < 0.05, *p* < 0.01, and *p* < 0.001.

## Results

### BBB Locomotor Scale

Initial assessment of SCI motor recovery was done using the BBB scale which has been shown to be informative in evaluating post-SCI function in rats (Basso et al., 1995). The score (0–21) represents different combinations of movements and the associated degree of recovery where an increased score represents better functional motor recovery.

**FIGURE 1:**
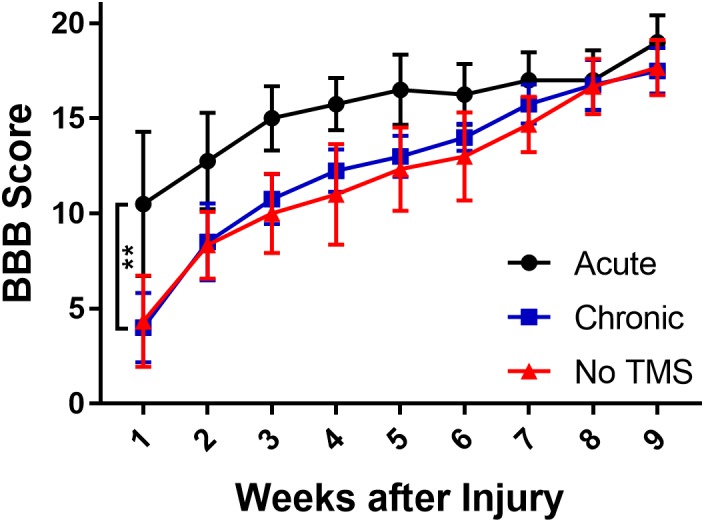
Graph showing effects of TMS treatment on SCI rats assessed by BBB scores over 9 weeks. BBB scores were assessed on a 21-point score (21 being a normal motor function). The scores show that the acute-TMS group (*n* = 4) showed significant improvement in scores over chronic (*p* = 0.004, *n* = 4) and no-TMS (*p* = 0.0015, *n* = 3) groups. The chronic-TMS group did not show significant improvement over the no-TMS group. Statistical analysis was performed with one-way ANOVA involving multiple comparisons.

We compared the change in the mean score between the TMS-treated groups (acute and chronic) and no-TMS group (Figure 1). The BBB score for the acute-TMS group (*n* = 4) had the best improvement amongst the three groups with a mean initial score of 10.5 ± 3.8 in the first week and continuous improvement leading to a mean score of 19 ± 1.14 in week 9. The chronic TMS group (*n* = 4) and the no-TMS group (*n* = 4) had similar BBB scores with steady improvement throughout the study. By the end of the 9th week all groups reached similar scores. We compared scores over the period of 9 weeks and report that the acute-TMS group had overall significance (*p* = 0.004) with a mean score of 15.53 ± 0.84 vs the chronic-TMS group (12.5 ± 1.41). The mean overall BBB score was also significant (*p* = 0.0015) for acute (15.53 ± 0.84) vs no-TMS group (12 ± 1.39). The comparison between the chronic vs the no-TMS group did not yield any statistical significance. This result showed that early intervention using TMS resulted in a better locomotor recovery following SCI.

### Gridwalk Test

**FIGURE 2:**
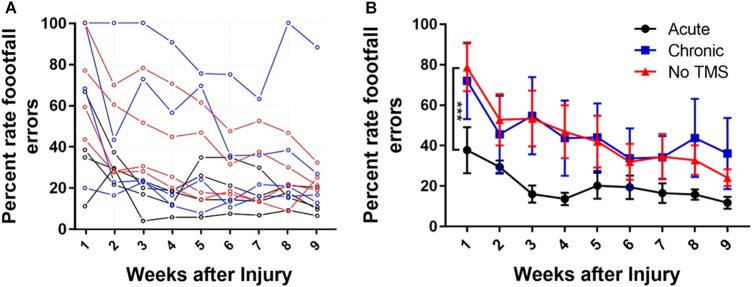
**(A)** Line graph showing mean gridwalk scores measured in terms of percent footfall errors across a period of 9 weeks for each animal across all groups. The acute-TMS group had the lowest percent footfall errors compared to the chronic and no-TMS group. **(B)** One-way ANOVA with multiple comparison analyses show significant improvement in percentage of footfall errors measured in acute (*n* = 4) vs chronic (*n* = 4, *p* = 0.0009) and acute vs no-TMS groups (*n* = 3, *p* = 0.0015). No significant differences were found between the chronic vs no-TMS groups across 9 weeks.

Data in (Figure 2A) show the percent footfall errors for each individual animal from all three groups over a period of 9 weeks. The acute-TMS group had the lowest percent footfall errors compared to the chronic and no-TMS groups. Many recovery processes show a rapid initial recovery phase followed by gradual slowing and approach a stable long-term level (asymptote), although this asymptote may not be reached during the short time of the experiment. Such processes are often fitted by an exponential decay curve. We fitted an exponential curve (data figure not shown) to the mean missed steps data from each group and tested whether the asymptotes differed between groups. Exponential curve fit equation is (*y* = a + be-t/τ, where “a” is the asymptotic recovery value, and τ is the recovery time constant in weeks and a + b = 100%). The estimates for the asymptotes (long-term values) for the three groups are 1. Acute-TMS 7.0 +/- 2.8; 2. No-TMS 34.9 +/- 5.8; 3. Chronic-TMS 26.4 +/- 3.3. The *p*-values for the differences between groups are: *P* < 0.005 when comparing 1 vs 3 (*t* = 4.48 on 6 df) and when comparing 1 vs 2 (*t* = 4.35 on 6 df).

In addition, we compared the percent of foot fall errors between the groups (Figure 2B) using multiple-comparison ANOVA analysis. Consistent with BBB score analysis, the acute-TMS rats had the lowest footfall error rate through 9 weeks with mean percent error rate of 37.75 ± 11.38 (Figure 2B) during the first week which consistently improved to 20.21 ± 6.3 in week 5 and 11.77 ± 2.9 at week 9. The acute-TMS group showed a significantly lowered foot fall error rate compared to chronic TMS (*p* = 0.0009) and no-TMS group (*p* = 0.0015). We report that the chronic- TMS group did not show significant differences in the footfall error rate compared to the no-TMS group.

### MEP Assessment

**FIGURE 3:**
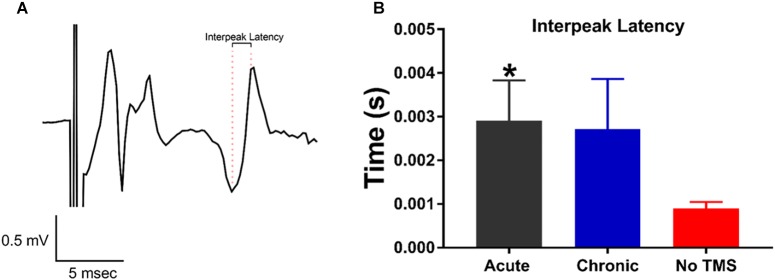
MEP responses recorded from the forepaws evoked by transcranial magnetic stimulation (TMS) pulses. **(A)** Representative MEP trace showing the distance in time between consecutive maximal peaks defined as inter-peak latency. **(B)** Data summary show a significant increase in inter-peak latency in rats treated with acute-TMS vs the no-TMS group (*p* = 0.018, denoted by ^∗^). The chronic vs. no-TMS group did not show significant differences in inter-peak latencies.

The MEP test is commonly used as a read-out of corticospinal excitability to probe the physiology of the motor cortex. Measuring MEPs provide quantification of this corticospinal excitability^,^ which TMS is known to modulate (Bestmann and Krakauer, 2015). Our goal was to investigate whether TMS can improve motor activity in the limbs following SCI. This was assessed through changes in the late-latency MEPs in the limbs. Inter-peak latency to evaluate the motor pathway integrity was calculated as the difference in time between the two consecutive peaks of the MEP (Figure 3A). For statistical analysis, late latency parameters were compared between acute, chronic and non-TMS treatment groups using a Student’s *t*-test. The inter-peak latencies for MEP calculated for both the right and left forelimbs were grouped together. Data graph (Figure 3B) show that the acute-TMS receiving rats have significantly longer inter-peak latencies (Mean ± SEM, 0.002908 ± 0.0009249, and *n* = 4) compared to no-TMS group (Mean ± SEM, 0.0009024 ± 0.000142, and *n* = 7). Though the chronic-TMS group had a longer inter-peak latency than the no-TMS group it did not show a significant difference.

### TMS Treatment Enhances the Evoked fMRI Signal

High resolution fMRI was used to measure somatosensory responses in SCI animals after TMS treatment. Evoked blood-oxygenation-level-dependent (BOLD) fMRI in the primary somatosensory cortex was measured in response to contralateral hind limb and forepaw stimulation 10 weeks post-SCI.

**FIGURE 4:**
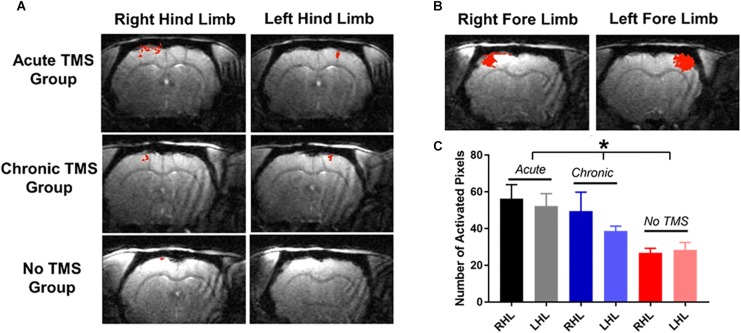
**(A)** fMRI measurements: were obtained 10 weeks after SCI. Individual fMRI cross-correlation statistical maps demonstrate increases in fMRI responses (in red) to contralateral stimulation of hindlimbs in the acute-TMS (*n* = 7) and chronic-TMS (*n* = 5) treatment groups, but not in the no- TMS group (*n* = 5). **(B)** Show sensory responses to stimulation of forepaws. **(C)** Data plots shows that the acute-TMS group demonstrated the greatest sensory responses to right and left hindlimb (LHL) stimulation compared to chronic-TMS and no-TMS groups. Statistical significance was determined by one-way ANOVA [*F*(5,28) = 3.554, *p* = 0.01)].

Our results demonstrate that the acute-TMS group (*n* = 7) showed the greatest increase in sensory responses evoked by stimulation of right hindlimb (RHL) with 56.29 ± 7.61 activated pixels, and left hindlimb (LHL) with 52.29 ± 6.73 activated pixels (Figure 4A). The chronic-TMS group had an intermediate response in both limbs and the sham control group failed to evoke any cortical response. Data analysis (Figure 4C) of sensory responses showed significantly larger response in terms of activated pixels in the acute-TMS group: 56.29 ± 7.61 (RHL) and 52.29 ± 6.73 (LHL). The chronic-TMS showed 49.4 ± 10.45 (RHL) and 38.6 ± 2.69 (LHL) activated pixels exhibiting moderate response. The no-TMS group showed very few activated pixels in response to stimulation: 26.8 ± 2.41 (RHL) and 28.4 ± 4.02 (LHL). One way ANOVA revealed significant differences in the means between the groups (*F*(5,28) = 3.554, *p* = 0.01). We also used *t*-test to report that the acute-TMS group was significantly larger than the no-TMS group (*p* = 0.0043). The chronic group also show an improved response over the no-TMS group (*p* = 0.02).

We also measured somatosensory responses to forepaw stimulation (Figure 4B), as the T9 contusion should not have affected evoked forepaw response. Indeed, the extent of the fMRI responses to left and right forepaw stimulating was similar to values that were reported previously (Li et al., 2011, 2014a,b; Han et al., 2013). The fMRI results showed that injured rats receiving TMS therapy immediately after the injury demonstrated the most extensive cortical activity in the weeks following injury.

## Discussion

### Non-invasive Brain Stimulation Improves Outlook in SCI

The aim of this study was to test whether applying TMS over the brain in minutes and weeks following SCI could facilitate recovery. In this study, rats subjected to SCI were assessed by behavior and physiological methods. Behavioral testing was evaluated by BBB score and gridwalk testing, and changes in corticospinal excitability was measured by MEP testing. Changes in cortical sensory information were evaluated using BOLD fMRI. TMS has been shown to be effective in facilitating recovery, enhancing neuroplasticity and alleviating pain in a number of neurological disorders and injuries (Lu et al., 2015; Shin et al., 2018) In addition, TMS has been shown to restore the transmission of corticospinal information and improve outcome in patients with SCI (Belci et al., 2004; Kumru et al., 2010).

There is a plethora of pharmacological and exercise therapy approaches in promoting brain plasticity after SCI. The use of 5-HT pharmacotherapy has been shown to promote cortical plasticity after SCI, especially in the improvement of behavioral outcome (Ganzer et al., 2013). Exercise interventions have also been shown to promote brain plasticity thereby aiding in the motor recovery in both the spinal cord and the brain. Animal studies involving exercise interventions have been shown to promote plasticity through the upregulation of various neurotrophic factors in the spinal cord (Liu et al., 2010; Keeler et al., 2012). In an animal model of traumatic brain injury we have recently demonstrated that combining enriched environment with TMS augment rehabilitation (Shin et al., 2018). Therefore, it is manifesting that a combination of therapies that target different physiological and neuroplasticity mechanisms might also be useful in facilitating recovery after SCI. A non-invasive method that changes cortical excitability thereby inducing plastic changes appears promising in the SCI arena (Gunduz et al., 2017).

### Immediate Intervention Improves Recovery

One of the goals of this study was to determine the timeframe for effective intervention. It has been shown in rats that cortical reorganization after SCI occur in several temporal stages. Long term cortical expansion assessed by fMRI 1 to 3 months after injury (Endo et al., 2007) is preceded by early changes in reorganization that occur within a week after injury (Sydekum et al., 2014). Early expansion of cortical representations has been shown to be associated with decreases in spine density in the deafferented cortical areas in rats (Kim et al., 2006). It has also been shown that a complete thoracic SCI in anesthetized rats immediately changes state of cortical activity (Yagüe et al., 2014). In addition, changes in cortical representation after SCI occur within hours using electrophysiological techniques (Humanes-Valera et al., 2013). This highlights the use of an early intervention strategy to mediate changes in cortical function that might benefit functional outcome.

An important factor to be taken into consideration for the intervention is the time elapsed after injury. The optimal window of time for initiating therapy, to promote recovery following SCI may be varied. Although studies using TMS were performed in the chronic phase of SCI (Kumru et al., 2010, 2016), this is the first report to evaluate the long-term effects of an acute-TMS application.

We report that changes in sensory perception are accompanied by increases in the motor outputs induced by acute TMS therapy. This is similar to previous research results that report that electrochemical neuromodulation restores motor activity in rodents with spinal cord contusion injury (Asboth et al., 2018). We also report that the beneficial effects of TMS in the acute group were seen earlier in the behavioral studies with all groups eventually catching up. Rapid improvement in the rehabilitation process is very impactful for human subjects, as this will reduce length of hospitalization, total cost of care, and minimize potential complications from a prolonged recovery process.

### Multimodal Methods to Quantify Behavioral, Motor and Sensory Outcomes

There are several behavioral tests in animal model that have been shown to be useful to monitor and evaluate functional outcome after SCI. These include the BBB scale, gridwalk test (Metz et al., 2000b; Dijkstra et al., 2006) plantar test (Hargreaves et al., 1988), and tail flick test. Although these tests are used frequently, they are highly variable and often do not have the sensitivity to monitor subtle and long-term changes. For example, the gridwalk test was shown to have a high degree of variability, due to the number of footfall errors correlating poorly with the BBB scoring system in rats (McEwen and Springer, 2006). In our study, the gridwalk and BBB scoring correlated well in terms of locomotor recovery. The BBB locomotion assessment of rat’s post-SCI, showed that the acute-TMS group recovered the fastest, with recovery plateauing by week 5. Additionally the acute-TMS group had a significantly better BBB score than the chronic and no-TMS group This is in agreement with previous evidence that show gradual recovery by week 5 (Nessler et al., 2006; Shinozaki et al., 2013). Gridwalk test results indicated that the acute-TMS group had the best scores with the fewest footfall errors compared to the chronic and no-TMS groups. We observed a trend of increased performance in BBB scoring and gridwalk test in the chronic-TMS group compared to no-TMS group although this trend did not reach significance. This suggests that TMS therapy could benefit if it starts at chronic stage after SCI injury (2 weeks post-SCI), but will be more effective if it begins in acute stages after the injury.

Previous studies have used MEP as a quantification method to assess recovery after SCI (Curt et al., 1998). In our study, the MEP test was performed to assess the extent of locomotor recovery post-TMS therapy. However, our results show that applying TMS to the sensorimotor cortex after SCI resulted in significantly longer inter-peak latency of MEPs. This may suggest a reorganization of motor pathways (Brum et al., 2016). As TMS is known to facilitate neuroplasticity, it is feasible that the applied TMS is facilitating reorganization of this corticospinal pathway as an adaptation to the global cortical silencing that occurs following SCI (Aguilar et al., 2010).

Various imaging techniques have been used successfully to monitor SCI changes. fMRI has been shown to be a useful tool in detecting changes in brain pathology after SCI (Wrigley et al., 2018) and brain connectivity, before and after SCI in mice (Matsubayashi et al., 2018). Furthermore, fMRI has been used to observe increased response to forepaw stimuli in the primary somatosensory region after thoracic SCI (Hofstetter et al., 2003). Here we used high-resolution fMRI obtained at ultra-high field of 11.7 T to monitor if TMS therapy induces post-injury neuroplasticity. We determined the optimal TMS therapy timing that lead to the greatest fMRI responses to limb stimulation. fMRI results from our study showed greater somatosensory response in the acute-TMS group followed by chronic with the no-TMS group showing very little signal. Our results show that fMRI is a sensitive method to report SCI neuroplasticity and report differences in cortical responses between the groups at 10 weeks post-injury while the behavioral tests suggested that, by the same time all the rats reached a similar level of behavioral performance.

Our study highlights the importance of utilizing a combination of methods that can assess behavioral performance, motor behavior, and sensory function in monitoring individuals post-SCI. Using fMRI as an additional tool alongside behavioral and electrophysiological methods provides a sensitive assessment of cortical function as well as a method to detect minute compensatory changes in response to brain activation.

We suggest that post-SCI TMS therapy should be further tested for its efficacy and safety. Subsequently, this non-invasive strategy could be readily translated to the clinic as an adjuvant to traditional rehabilitation strategies. One of the limitations of our study is the use of general anesthesia during TMS treatments electrophysiology, and fMRI measurements in the animal model, which may not mimic the human condition. However, the insights gained from this and similar animal model studies are valuable in developing and evaluating new therapies. Moreover, our results suggest that early intervention is key for recovery and might play a role in medical strategies and trauma care interventions.

## Ethics Statement

All animal procedures were conducted in accordance with the NIH Guide for the Care and Use of Laboratory Animals and approved by the Johns Hopkins University Animal Care and Use Committee.

## Author Contributions

VK and GP designed all experiments and wrote the manuscript. VK, SS, and VB performed the experiments. GP, VK, SS, KS, PC, and MR analyzed the data. All authors read and approved the final manuscript.

## Conflict of Interest Statement

The authors declare that the research was conducted in the absence of any commercial or financial relationships that could be construed as a potential conflict of interest.

## References

[B1] Aguilar J. Humanes-Valera D. Alonso-Calvino E. Yague J. G. Moxon K. A. Oliviero A. (2010). Spinal cord injury immediately changes the state of the brain. *J. Neurosci.* 30 7528–7537. 10.1523/JNEUROSCI.0379-10.2010 20519527 PMC3842476

[B2] Anderson K. D. Sharp K. G. Steward O. (2009). Bilateral cervical contusion spinal cord injury in rats. *Exp. Neurol.* 220 9–22. 10.1016/j.expneurol.2009.06.012 19559699 PMC2761499

[B3] Asboth L. Friedli L. Beauparlant J. Martinez-Gonzalez C. Anil S. Rey E. (2018). Cortico-reticulo-spinal circuit reorganization enables functional recovery after severe spinal cord contusion. *Nat. Neurosci.* 21 576–588. 10.1038/s41593-018-0093-5 29556028

[B4] Basso D. M. Beattie M. S. Bresnahan J. C. (1995). A sensitive and reliable locomotor rating scale for open field testing in rats. *J. Neurotrauma* 12 1–21. 10.1089/neu.1995.12.1 7783230

[B5] Belci M. Catley M. Husain M. Frankel H. Davey N. (2004). Magnetic brain stimulation can improve clinical outcome in incomplete spinal cord injured patients. *Spinal Cord* 42:417. 10.1038/sj.sc.3101613 15111994

[B6] Benito J. Kumru H. Murillo N. Costa U. Medina J. Tormos J. (2012). Motor and gait improvement in patients with incomplete spinal cord injury induced by high-frequency repetitive transcranial magnetic stimulation. *Top. Spinal Cord Injury Rehabil.* 18 106–112. 10.1310/sci1802-106 23459246 PMC3584757

[B7] Bestmann S. Krakauer J. W. (2015). The uses and interpretations of the motor-evoked potential for understanding behaviour. *Exp. Brain Res.* 233 679–689. 10.1007/s00221-014-4183-7 25563496

[B8] Boland R. A. Lin C. S.-Y. Engel S. Kiernan M. C. (2010). Adaptation of motor function after spinal cord injury: novel insights into spinal shock. *Brain* 134 495–505. 10.1093/brain/awq289 20952380

[B9] Bonatz H. Röhrig S. Mestres P. Meyer M. Giehl K. M. (2000). An axotomy model for the induction of death of rat and mouse corticospinal neurons in vivo. *J. Neurosci. Methods* 100 105–115. 10.1016/s0165-0270(00)00238-7 11040372

[B10] Brum M. Cabib C. Valls-Solé J. (2016). Clinical value of the assessment of changes in Mep duration with voluntary contraction. *Front. Neurosci.* 9:505. 10.3389/fnins.2015.00505 26793051 PMC4707281

[B11] Cao Q. Zhang Y. P. Iannotti C. Devries W. H. Xu X.-M. Shields C. B. (2005). Functional and electrophysiological changes after graded traumatic spinal cord injury in adult rat. *Exp. Neurol.* 191 S3–S16. 15629760 10.1016/j.expneurol.2004.08.026

[B12] Courtine G. Gerasimenko Y. Van Den Brand R. Yew A. Musienko P. Zhong H. (2009). Transformation of nonfunctional spinal circuits into functional states after the loss of brain input. *Nat. Neurosci.* 12:1333. 10.1038/nn.2401 19767747 PMC2828944

[B13] Curt A. Keck M. E. Dietz V. (1998). Functional outcome following spinal cord injury: significance of motor-evoked potentials and ASIA scores. *Arch. Phys. Med. Rehabil.* 79 81–86. 10.1016/s0003-9993(98)90213-1 9440423

[B14] Dijkstra S. Duis S. Pans I. Lankhorst A. Hamers F. Veldman H. (2006). Intraspinal administration of an antibody against CD81 enhances functional recovery and tissue sparing after experimental spinal cord injury. *Exp. Neurol.* 202 57–66. 10.1016/j.expneurol.2006.05.011 16806185

[B15] Endo T. Spenger C. Tominaga T. Brene S. Olson L. (2007). Cortical sensory map rearrangement after spinal cord injury: fMRI responses linked to Nogo signalling. *Brain* 130 2951–2961. 10.1093/brain/awm237 17913768

[B16] Formento E. Minassian K. Wagner F. Mignardot J. B. Le Goff-Mignardot C. G. Rowald A. (2018). Electrical spinal cord stimulation must preserve proprioception to enable locomotion in humans with spinal cord injury. *Nat. Neurosci.* 21:1728. 10.1038/s41593-018-0262-6 30382196 PMC6268129

[B17] Ganzer P. D. Moxon K. A. Knudsen E. B. Shumsky J. S. (2013). Serotonergic pharmacotherapy promotes cortical reorganization after spinal cord injury. *Exp. Neurol.* 241 84–94. 10.1016/j.expneurol.2012.12.004 23262119 PMC4470269

[B18] Ghosh A. Haiss F. Sydekum E. Schneider R. Gullo M. Wyss M. T. (2010). Rewiring of hindlimb corticospinal neurons after spinal cord injury. *Nat. Neurosci.* 13:97. 10.1038/nn.2448 20010824

[B19] Ghosh A. Peduzzi S. Snyder M. Schneider R. Starkey M. Schwab M. E. (2012). Heterogeneous spine loss in layer 5 cortical neurons after spinal cord injury. *Cereb Cortex* 22 1309–1317. 10.1093/cercor/bhr191 21840844

[B20] Gunduz A. Rothwell J. Vidal J. Kumru H. (2017). Non-invasive brain stimulation to promote motor and functional recovery following spinal cord injury. *Neural Regen. Res.* 12:1933. 10.4103/1673-5374.221143 29323025 PMC5784334

[B21] Han Y. Li N. Zeiler S. R. Pelled G. (2013). Peripheral nerve injury induces immediate increases in layer v neuronal activity. *Neurorehabil. Neural Repair* 27 664–672. 10.1177/1545968313484811 23599222 PMC3729632

[B22] Hargreaves K. Dubner R. Brown F. Flores C. Joris J. (1988). A new and sensitive method for measuring thermal nociception in cutaneous hyperalgesia. *Pain* 32 77–88. 10.1016/0304-3959(88)90026-7 3340425

[B23] Harkema S. Gerasimenko Y. Hodes J. Burdick J. Angeli C. Chen Y. (2011). Effect of epidural stimulation of the lumbosacral spinal cord on voluntary movement, standing, and assisted stepping after motor complete paraplegia: a case study. *Lancet* 377 1938–1947. 10.1016/S0140-6736(11)60547-3 21601270 PMC3154251

[B24] Hofstetter C. P. Schweinhardt P. Klason T. Olson L. Spenger C. (2003). Numb rats walk–a behavioural and fMRI comparison of mild and moderate spinal cord injury. *Eur. J. Neurosci.* 18 3061–3068. 10.1111/j.1460-9568.2003.03062.x 14656301

[B25] Humanes-Valera D. Aguilar J. Foffani G. (2013). Reorganization of the intact somatosensory cortex immediately after spinal cord injury. *PLoS One* 8:e69655. 10.1371/journal.pone.0069655 23922771 PMC3726757

[B26] Jain N. Qi H.-X. Collins C. E. Kaas J. H. (2008). Large-scale reorganization in the somatosensory cortex and thalamus after sensory loss in macaque monkeys. *J. Neurosci.* 28 11042–11060. 10.1523/jneurosci.2334-08.2008 18945912 PMC2613515

[B27] Jazayeri S. B. Beygi S. Shokraneh F. Hagen E. M. Rahimi-Movaghar V. (2015). Incidence of traumatic spinal cord injury worldwide: a systematic review. *Eur. Spine J.* 24 905–918. 10.1007/s00586-014-3424-6 24952008

[B28] Keeler B. E. Liu G. Siegfried R. N. Zhukareva V. Murray M. Houlé J. D. (2012). Acute and prolonged hindlimb exercise elicits different gene expression in motoneurons than sensory neurons after spinal cord injury. *Brain Res.* 1438 8–21. 10.1016/j.brainres.2011.12.015 22244304 PMC3273584

[B29] Kim B. G. Dai H.-N. Mcatee M. Vicini S. Bregman B. S. (2006). Remodeling of synaptic structures in the motor cortex following spinal cord injury. *Exp. Neurol.* 198 401–415. 10.1016/j.expneurol.2005.12.010 16443221

[B30] Kumru H. Benito-Penalva J. Valls-Sole J. Murillo N. Tormos J. M. Flores C. (2016). Placebo-controlled study of rTMS combined with Lokomat^®^ gait training for treatment in subjects with motor incomplete spinal cord injury. *Exp. Brain Res.* 234 3447–3455. 10.1007/s00221-016-4739-9 27469242

[B31] Kumru H. Murillo N. Vidal Samso J. Valls-Sole J. Edwards D. Pelayo R. (2010). Reduction of spasticity with repetitive transcranial magnetic stimulation in patients with spinal cord injury. *Neurorehabil. Neural Repair* 24 435–441. 10.1177/1545968309356095 20053952 PMC3366152

[B32] Kuppuswamy A. Balasubramaniam A. Maksimovic R. Mathias C. Gall A. Craggs M. (2011). Action of 5 Hz repetitive transcranial magnetic stimulation on sensory, motor and autonomic function in human spinal cord injury. *Clin. Neurophysiol.* 122 2452–2461. 10.1016/j.clinph.2011.04.022 21600843

[B33] Lee M. Kiernan M. C. Macefield V. G. Lee B. B. Lin C. S.-Y. (2015). Short-term peripheral nerve stimulation ameliorates axonal dysfunction after spinal cord injury. *J. Neurophysiol.* 113 3209–3218. 10.1152/jn.00839.2014 25787956 PMC4432679

[B34] Lefaucheur J.-P. André-Obadia N. Antal A. Ayache S. S. Baeken C. Benninger D. H. (2014). Evidence-based guidelines on the therapeutic use of repetitive transcranial magnetic stimulation (rTMS). *Clin. Neurophysiol.* 125 2150–2206. 25034472 10.1016/j.clinph.2014.05.021

[B35] Li N. Downey J. E. Bar-Shir A. Gilad A. A. Walczak P. Kim H. (2011). Optogenetic-guided cortical plasticity after nerve injury. *Proc. Natl. Acad. Sci.* 108 8838–8843. 10.1073/pnas.1100815108 21555573 PMC3102379

[B36] Li N. Van Zijl P. Thakor N. Pelled G. (2014a). Study of the spatial correlation between neuronal activity and BOLD fMRI responses evoked by sensory and channelrhodopsin-2 stimulation in the rat somatosensory cortex. *J. Mol. Neurosci.* 53 553–561. 10.1007/s12031-013-0221-3 24443233 PMC4104155

[B37] Li N. Yang Y. Glover D. P. Zhang J. Saraswati M. Robertson C. (2014b). Evidence for impaired plasticity after traumatic brain injury in the developing brain. *J. Neurotrauma* 31 395–403. 10.1089/neu.2013.3059 24050267 PMC3922417

[B38] Liu G. Keeler B. E. Zhukareva V. Houlé J. D. (2010). Cycling exercise affects the expression of apoptosis-associated microRNAs after spinal cord injury in rats. *Exp. Neurol.* 226 200–206. 10.1016/j.expneurol.2010.08.032 20816819 PMC2963016

[B39] Lu H. Kobilo T. Robertson C. Tong S. Celnik P. Pelled G. (2015). Transcranial magnetic stimulation facilitates neurorehabilitation after pediatric traumatic brain injury. *Sci. Rep.* 5:14769. 10.1038/srep14769 26440604 PMC4594036

[B40] Martin R. Sadowsky C. Obst K. Meyer B. Mcdonald J. (2012). Functional electrical stimulation in spinal cord injury: from theory to practice. *Top. Spinal Cord Injury Rehabil.* 18 28–33. 10.1310/sci1801-28 23459150 PMC3584753

[B41] Matsubayashi K. Nagoshi N. Komaki Y. Kojima K. Shinozaki M. Tsuji O. (2018). Assessing cortical plasticity after spinal cord injury by using resting-state functional magnetic resonance imaging in awake adult mice. *Sci. Rep.* 8:14406. 10.1038/s41598-018-32766-8 30258091 PMC6158265

[B42] McEwen M. L. Springer J. E. (2006). Quantification of locomotor recovery following spinal cord contusion in adult rats. *J. Neurotrauma* 23 1632–1653. 10.1089/neu.2006.23.1632 17115910

[B43] Metz G. A. Curt A. Van De Meent H. Klusman I. Schwab M. E. Dietz V. (2000a). Validation of the weight-drop contusion model in rats: a comparative study of human spinal cord injury. *J. Neurotrauma* 17 1–17. 10.1089/neu.2000.17.1 10674754

[B44] Metz G. A. Merkler D. Dietz V. Schwab M. E. Fouad K. (2000b). Efficient testing of motor function in spinal cord injured rats. *Brain Res.* 883 165–177. 10.1016/s0006-8993(00)02778-5 11074045

[B45] Nakamae T. Tanaka N. Nakanishi K. Fujimoto Y. Sasaki H. Kamei N. (2010). Quantitative assessment of myelopathy patients using motor evoked potentials produced by transcranial magnetic stimulation. *Eur. Spine J.* 19 685–690. 10.1007/s00586-009-1246-8 20033461 PMC2899952

[B46] Nessler J. A. Leon R. D. D. Sharp K. Kwak E. Minakata K. Reinkensmeyer D. J. (2006). Robotic gait analysis of bipedal treadmill stepping by spinal contused rats: characterization of intrinsic recovery and comparison with Bbb. *J. Neurotrauma* 23 882–896. 10.1089/neu.2006.23.882 16774473

[B47] O’Reardon J. P. Solvason H. B. Janicak P. G. Sampson S. Isenberg K. E. Nahas Z. (2007). Efficacy and safety of transcranial magnetic stimulation in the acute treatment of major depression: a multisite randomized controlled trial. *Biol. Psychiatry* 62 1208–1216. 10.1016/j.biopsych.2007.01.018 17573044

[B48] Pelled G. Bergstrom D. A. Tierney P. L. Conroy R. S. Chuang K. H. Yu D. (2009). Ipsilateral cortical fmri responses after peripheral nerve damage in rats reflect increased interneuron activity. *Proc. Natl. Acad. Sci. U.S.A.* 106 14114–14119. 10.1073/pnas.0903153106 19666522 PMC2720851

[B49] Pelled G. Dodd S. J. Koretsky A. P. (2006). Catheter confocal fluorescence imaging and functional magnetic resonance imaging of local and systems level recovery in the regenerating rodent sciatic nerve. *Neuroimage* 30 847–856. 10.1016/j.neuroimage.2005.10.027 16343952

[B50] Scheff S. W. Rabchevsky A. G. Fugaccia I. Main J. A. Lumpp J. E. Jr. (2003). Experimental modeling of spinal cord injury: characterization of a force-defined injury device. *J. Neurotrauma* 20 179–193. 10.1089/08977150360547099 12675971

[B51] Schulz R. Gerloff C. Hummel F. C. (2013). Non-invasive brain stimulation in neurological diseases. *Neuropharmacology* 64 579–587. 10.1016/j.neuropharm.2012.05.016 22687520

[B52] Shin S. S. Krishnan V. Stokes W. Robertson C. Celnik P. Chen Y. (2018). Transcranial magnetic stimulation and environmental enrichment enhances cortical excitability and functional outcomes after traumatic brain injury. *Brain Stimul.* 11 1306–1313. 10.1016/j.brs.2018.07.050 30082198 PMC6204305

[B53] Shin S. S. Pelled G. (2017). Novel neuromodulation techniques to assess interhemispheric communication in neural injury and neurodegenerative diseases. *Front. Neural Circ.* 11:15. 10.3389/fncir.2017.00015 28337129 PMC5343068

[B54] Shinozaki M. Yasuda A. Nori S. Saito N. Toyama Y. Okano H. (2013). Novel method for analyzing locomotor ability after spinal cord injury in rats. *Neurol. Med. Chir.* 53 907–913. 10.2176/nmc.tn2012-0223 24097095 PMC4508735

[B55] Singh A. Tetreault L. Kalsi-Ryan S. Nouri A. Fehlings M. G. (2014). Global prevalence and incidence of traumatic spinal cord injury. *Clin. Epidemiol.* 6:309. 10.2147/CLEP.S68889 25278785 PMC4179833

[B56] Sydekum E. Ghosh A. Gullo M. Baltes C. Schwab M. Rudin M. (2014). Rapid functional reorganization of the forelimb cortical representation after thoracic spinal cord injury in adult rats. *Neuroimage* 87 72–79. 10.1016/j.neuroimage.2013.10.045 24185021

[B57] Van De Meent H. Hosman A. J. Hendriks J. Zwarts M. Group E.-S. S. Schubert M. (2010). Severe degeneration of peripheral motor axons after spinal cord injury: a European multicenter study in 345 patients. *Neurorehabil. Neural Repair* 24 657–665. 10.1177/1545968310368534 20439500

[B58] Wrigley P. J. Siddall P. J. Gustin S. M. (2018). New evidence for preserved somatosensory pathways in complete spinal cord injury: a fMRI study. *Hum. Brain Mapp.* 39 588–598. 10.1002/hbm.23868 29080262 PMC6866574

[B59] Yagüe J. Humanes-Valera D. Aguilar J. Foffani G. (2014). Functional reorganization of the forepaw cortical representation immediately after thoracic spinal cord hemisection in rats. *Exp. Neurol.* 257 19–24. 10.1016/j.expneurol.2014.03.015 24685666

